# Network analysis of team communication in a busy emergency department

**DOI:** 10.1186/1472-6963-13-109

**Published:** 2013-03-22

**Authors:** P Daniel Patterson, Anthony J Pfeiffer, Matthew D Weaver, David Krackhardt, Robert M Arnold, Donald M Yealy, Judith R Lave

**Affiliations:** 1 Department of Emergency Medicine, School of Medicine, University of Pittsburgh, Pittsburgh, Pennsylvania, USA; 2 Heinz School of Public Policy and Management, Carnegie Mellon University, Pittsburgh, Pennsylvania, USA; 3 Department of Medicine, School of Medicine, University of Pittsburgh, Pittsburgh, Pennsylvania, USA; 4 Department of Health Policy and Management, Graduate School of Public Health, University of Pittsburgh, Pittsburgh, Pennsylvania, USA

**Keywords:** Teamwork, Communication, Social network analysis, Emergency medicine

## Abstract

**Background:**

The Emergency Department (ED) is consistently described as a high-risk environment for patients and clinicians that demands colleagues quickly work together as a cohesive group. Communication between nurses, physicians, and other ED clinicians is complex and difficult to track. A clear understanding of communications in the ED is lacking, which has a potentially negative impact on the design and effectiveness of interventions to improve communications. We sought to use Social Network Analysis (SNA) to characterize communication between clinicians in the ED.

**Methods:**

Over three-months, we surveyed to solicit the communication relationships between clinicians at one urban academic ED across all shifts. We abstracted survey responses into matrices, calculated three standard SNA measures (network density, network centralization, and in-degree centrality), and presented findings stratified by night/day shift and over time.

**Results:**

We received surveys from 82% of eligible participants and identified wide variation in the magnitude of communication cohesion (density) and concentration of communication between clinicians (centralization) by day/night shift and over time. We also identified variation in in-degree centrality (a measure of power/influence) by day/night shift and over time.

**Conclusions:**

We show that SNA measurement techniques provide a comprehensive view of ED communication patterns. Our use of SNA revealed that frequency of communication as a measure of interdependencies between ED clinicians varies by day/night shift and over time.

## Background

Poor communication between health care teammates is a key factor in medical error [1-3]. The Joint Commission and other leading authorities of quality and safety in the United States identified communication lapses as responsible for a large proportion of poor patient and provider outcomes [4,5]. Improving communication between teammates is a central theme in safety improvement in healthcare, aviation, and other high-risk industries [6,7].

Communication between care providers in the Emergency Department (ED) is essential for the delivery of safe and effective care [8]. A minimum of 19 complex communication events occur per patient in the ED, with complex cases resulting in much greater frequency of communication [9]. The communication occurring between patient and clinician and between clinicians is threatened by the fast pace and the often unpredictable nature of patient need and demand [10]. One study determined that one third of all communications between clinician teammates are interrupted and 10% of all communications involve care decisions for more than one patient simultaneously [11]. This results in the frequent loss of critical patient care information [1,10,12-15]. Left alone, this form of team communication may impede the flow of key patient information and increase the threat of a poor safety outcome for patient and clinician.

We have only just begun to develop and test measures of communication for the ED setting. Our continued interest in ED-based team communication is supported by expert opinion that team communication underpins all components of teamwork and provides a unique “window into team cognition” and behavior [16]. Analysis of team communication provides “a rich source of information about a team’s shared understanding” of individual roles, tasks, and team goals [17]. However, measurement of team communication is complex [18]. Identifying the best method for communication analysis, especially in the ED, is an obstacle to investigating team communication and performance [16].

A systems or network approach is one method to study team communication. Under this approach, investigators measure the frequency of communication between teammates in an organization [19]. Recent research applying a systems or network approach includes link analysis used by human factors engineers, direct observation, self-report, and Social Network Analysis (SNA) [20-24]. Among these, SNA is unique in that it graphically depicts patterns of teammate communication and interaction. SNA also includes a set of statistics that can quantify the magnitude of communication between teammates [21,23,25,26]. Investigators have found SNA useful in healthcare settings because it can provide visualization of communication patterns not clearly detectable from link analysis, direct observation, or other techniques that primarily focus on communication among a subset of teammates rather than all teammates [21,25,26]. These visualizations include uncovering cliques where all types of communication (regardless of content) may be concentrated in small groups [22,27,28]. SNA visualizations detect paths where communication flow is channeled, abruptly ends, or is restricted [22,27,28]. Previous research has used SNA graphs to identify individual team members that receive or produce the most or least communication in the workplace not easily detected using other techniques [22,27,28]. SNA statistics complement visualizations by quantifying the structural characteristics of communication patterns between teammates. Investigators may use these statistics to make comparisons of communication patterns across time, pre/post intervention, across different groups of teammates, and across different units within and outside organizations.

The aim of this study was to use SNA techniques to characterize patterns of communication among all clinicians and staff employed in a busy academic ED over multiple time points and time of day. Our use of SNA in the ED setting may be instructive to others seeking to evaluate communication patterns and safety in the ED setting.

## Methods

### Study setting and design

We used a case study design of one large academic ED with a patient volume approximately 54,000 visits annually. The hospital is located in an urban center surrounded by multiple university campuses in a county with 1.2 million residents. We used data from surveys to measure communication relationships among ED clinicians and non-clinician staff. The University of Pittsburgh Institutional Review Board approved this study as exempt on 12/15/2008 (IRB reference number PRO08120218).

We used SNA survey techniques to describe the patterns of communications between clinical and support personnel employed in this ED. We chose this method based on the following: 1) SNA views behaviors and interactions between individuals as interdependent rather than independent; 2) communication and interactions between individuals depend on the workplace environment; 3) SNA techniques help visualize the interdependencies, relationships, and communication between individuals revealing patterns and quantitative measures of pattern structure; 4) these patterns and measures can identify individuals or groups of individuals that are isolated, overburdened, facilitators, negotiators, associated with cliques, or are powerful brokers of information dissemination; 5) SNA surveys workers to obtain the data for documenting interactions and communication between colleagues in the workplace; and 6) SNA is viewed as a valuable tool in designing methods for improving quality and safety in healthcare [22,27-30].

### Instrument

Team communication is often investigated by documenting frequency of interactions between individuals [31]. Currently, there are a limited number of reliable and valid measurement tools that have been appropriately contextualized for the ED setting. We developed a three-item SNA survey by adapting items used in a previous ED study to measure frequency of communication between teammates [23]. We followed standard SNA practice and presented a single survey item on its own page and included a standard item stem: “*Think only about this shift. Record below the total number of times you initiated communication*…” The item stem was followed by one of three statements: 1) …*when you had a general problem that you needed to solve*. 2) …*when you needed advice about medication*. 3) …*when you wanted to generally socialize*. Each SNA survey included the names of all eligible ED personnel on the shift in question. Respondents indicated their response on a 10-point Likert scale that ranged from 0 to 10+. We assigned labels to scale anchors and the midpoint: 0 as “*none;*” option 5 as “*about 5 times;*” and option 10+ as “*more than 10*.”

### Study protocol

We obtained a list of the names of all ED clinicians and non-clinician staff from department administrators and populated paper-based SNA surveys with these names prior to each targeted shift. The shift length of physicians, nurses, and other clinicians varies with some clinicians leaving earlier or later than others. Most clinicians work 8 or 12 hour shifts and begin their shift between the hours of 0500 and 0900 or 1500 and 1900. To maximize recruitment, we positioned a co-investigator in the ED from 0500–0900 hours for night shift data collection and 1500–1900 hours for day-shift data collection. We limited data collection to these time blocks to avoid disrupting normal operations and patient care. We surveyed both night and day shift workers to address the potential for differences in communication between colleagues based on known differences in patient volume with greater than 60% of all visits expected to occur during night shift hours [32]. We performed multiple surveys at least once each week (the Tuesday overnight shift and Wednesday day shift) over three consecutive months in 2009 to address concerns that a one-time assessment may over or underestimate measures of communication. We chose this time period and method of survey collection because: 1) We wanted to collect SNA data over multiple time points; and 2) Administrators indicated conducting the surveys during these time periods would minimize disruption of ED operations.

**Figure 1:**
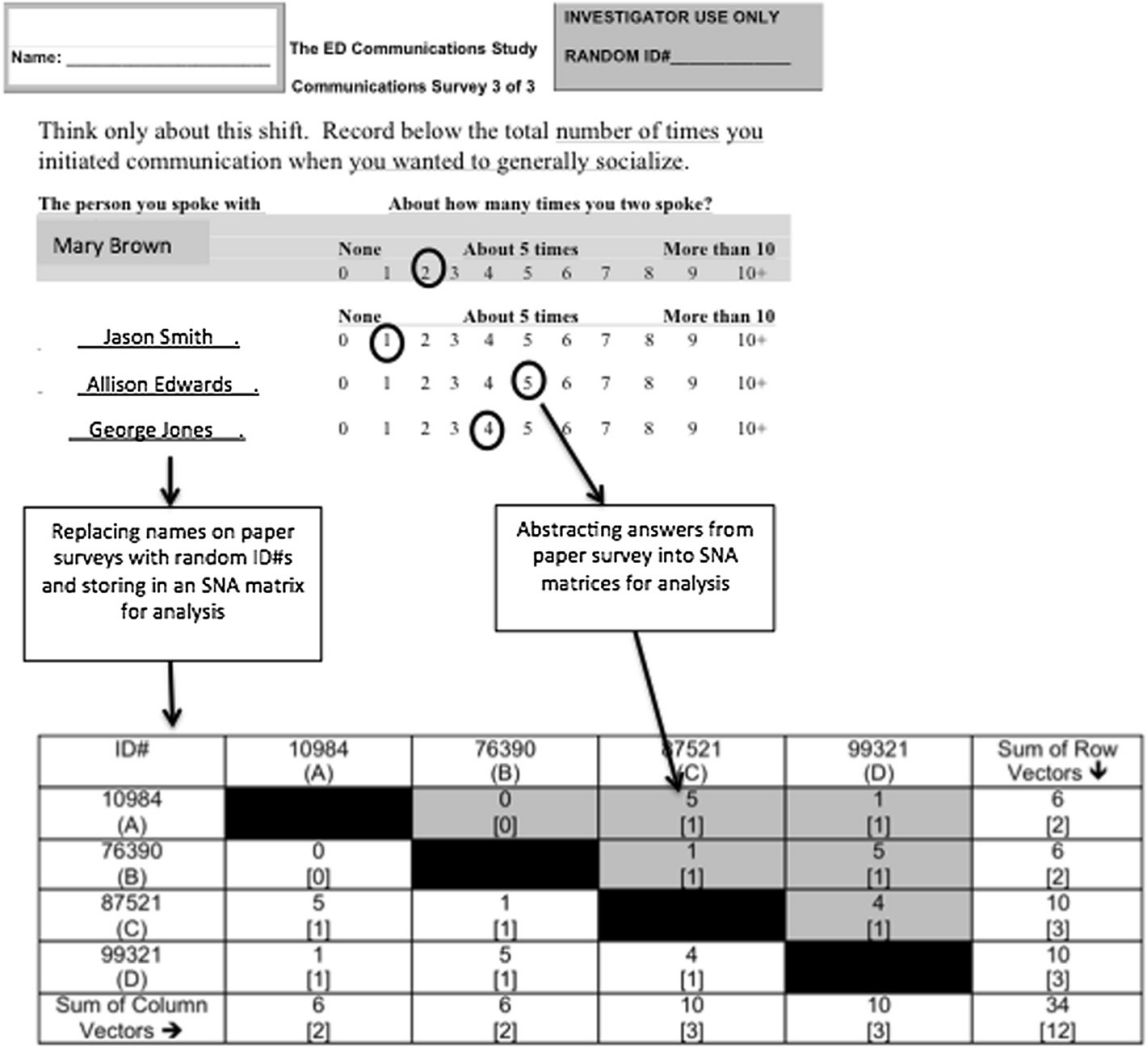
Illustration of SNA survey and matrices used for calculation of SNA measures.

Participation was voluntary for all clinicians and non-clinician staff. ED clinicians included attending physicians (AMD), emergency medicine residents (RMD), staff nurses (SRN), triage nurses (TRN), trauma nurses (TRAMRN), charge nurses (CRN), patient care technicians (PCT), and health unit coordinators (HUC). The registration clerk (RC) was the only non-clinician staff member. We excluded all non-emergency medicine residents and other personnel given their sporadic presence. Figure 1 illustrates our process for de-identification and abstraction of paper-based survey data into SNA matrices.

### Analysis of data

We used frequencies, percentages, means, and standard deviations to describe characteristics of participants in our study. We calculated three standard SNA measures to characterize the structural patterns of communication flow: Network Density, Network Centralization, and In-Degree Centralization.

*Network density* is the most widely used SNA measure [28]. Network density measures how close-knit the members of the network are and is often referred to as an overall measure of interaction. Density is the proportion of existing communication relationships between members (presence or absence) at the dyadic level divided by the total possible number of communication ties at the dyad level [28]. Values for network density range from 0 to 100 with higher values indicating greater cohesion and frequent communication among all members in a defined network [28]. In Figure 1, density would be 83.3%, which is calculated using the numbers located within the brackets [#] on one side of the diagonal of the table (see shaded area). The calculation involves dividing the number of dyads in the matrix (n=5) by all possible dyads, which are six (5/6 = 83.3*%*). Experts believe an organization that is completely cohesive (has a density score close to 100) is an organization where coworkers (teammates) possess the capability to coordinate efficiently and effectively to meet the needs of one another [29]. An organization with low density measures (near 0) suggest coworkers may lack experience or familiarity with one another, coordination between coworkers may be limited, and time to completion of required tasks may be extended compared to high-density settings [33-36]. Odds of high density may be greater in smaller workplaces where the number of colleagues to connect to is limited. No matter the size, it is often rare for a workplace to have ties between all colleagues [28,37].

*Network centralization* refers to the concentration of communication in the network and is analogous to variance (a measure of dispersion) [27]. Network centralization is the sum of the maximum local point degree centrality minus the local point centrality for each vector divided by the maximum possible value for local point centrality [28]. The calculation can be described in three steps using the example data in Figure 1. First, subtract the number of communication relationships between each teammate (presence/absence) and the one teammate with the highest frequency of communication: [C-D= 3-3=0; C-A= 3-2=1; C-B=3-2=1]. Sum the differences to obtain the numerator [0 + 1 + 1 = 2]. Next, solve for the denominator by calculating maximum possible centrality beginning with C holding the maximum value: A=1, B=1, C=3, and D=1. Subtract each person’s measure by the maximum [C-A= 3-1=2; C-B=3-1=2; and C-D=3-1=2 with the sum 2+2+2 equal to 6. Finally, solve for network centrality by dividing numerator by denominator [2/6 = 0.33] and express as a percentage = 33.33%. Network centralization values range from 0 to 100. Network density and centralization are complementary measures [37]. Low centralization (or “decentralization”) indicates greater distribution of communication across teammates with no single team member enjoying a high level of communication over any other team member in the network. A decentralized network could be interpreted as a highly communicative network of teammates, where information is communicated frequently between all in the team. In contrast, higher values of network centralization indicate that communication is concentrated to one or a select few teammates in the team, leaving some teammates isolated or “out of the loop.”

*In degree centrality*, which is related to network centralization, is commonly used to rank individuals based on their positioning/influence in the team / network [28]. Investigators have used network centralization and in-degree centrality to identify the important and influential individuals within organizations [37,38]. A teammate can be influential in several ways. He/she may be the person providing/initiating the communication to his/her teammates (out-going communication) and thus the source of valuable information for the team. A person may be influential because he/she is the recipient/target of communication from many other teammates (in-coming communication) due to his/her role in the organizational hierarchy or due to his/her reputation as a valuable source of information. In-degree centrality (in-coming communication) is the sum of communication ties from all teammates in the network standardized by dividing the sum by all possible communication relationships [28]. An individual is considered prominent, important, or powerful when he/she has a high level of in-degree centrality [28].

While the three relations are conceptually independent, there is evidence that people often choose to approach others for technical advice those with whom they have positive affective ties [39]. That is, in practice, these relations may overlap to a considerable degree. To determine the extent to which these relations may indeed overlap, perhaps even to the point of being redundant, we performed pair-wise correlations between each of the networks (general problem solving, advice about medication, and general social exchanges). Also, late night shifts may put different technical and social demands on the participants. Thus, we examined the correlations among these networks in the day shifts and the evening shifts separately to see if different overlapping patterns emerged during these two distinct time periods.

It is well known that network data such as we have in this study are not comprised of independent observations [28]. This lack of independence in network data has been shown to severely bias significance tests of relationships between networks [40], the very question we want to explore here. To address this problem, we used nonparametric bivariate QAP tests to determine which relations were indeed significantly related to each other [41]. The QAP test is a restricted permutation test, wherein each permutation preserves the auto-correlational structure of the observed data. The QAP test has been shown to be robust against this nagging problem of autocorrelation in the network data, removing virtually all the bias that would be introduced with more traditional statistical tests [40,42]. We used R to perform QAP analyses across all data collection time points and followed standard procedures outlined in previous research [41]. We used UCINET Version 6.205 to calculate density, network centralization, and in-degree centrality [43].

## Results

We received 336 SNA surveys completed by 103 unique respondents, with 70 of those respondents completing more than one survey. The completed surveys represent 82% of all possible SNA surveys from eligible respondents. Participation varied across shifts and time point and across roles (Tables 1 &2). Among participants (n=103), the mean age was 35 years, the mean years of ED experience was 5, and mean years in healthcare 11. The most common level of education among participants was some college or an associates or undergraduate degree (63.4%).

**Table 1:** Response rates by night/day and week of study period

	**Survey completed**	**Did not complete survey**		
**Shift / Date**	**Person scheduled and in the ED during survey period (B)**	**Person refused to complete survey (C)**	**Person in the ED but not located to complete survey (D)**	**Response rate** [**B** ÷ (**B** + **C** + **D**)]
**May 6 AM**	10	1	3	71.4%
**May 6 PM**	17	1	2	85%
**May 13 AM**	8	1	0	88.9%
**May 13 PM**	11	4	3	61.1%
**May 20 AM**	11	2	0	84.6%
**May 20 PM**	7	9	0	43.7%
**May 27 AM**	13	0	0	100%
**May 27 PM**	14	5	1	70%
**June 3 AM**	10	1	0	91%
**June 3 PM**	13	4	2	68.4%
**June 10 AM**	14	1	0	93.3%
**June 10 PM**	19	3	0	86.4%
**June 17 AM**	14	0	0	100%
**June 17 PM**	18	3	0	85.7%
**June 24 AM**	14	1	0	93.3%
**June 24 PM**	18	3	0	85.7%
**July 1 AM**	14	1	0	93.3%
**July 1 PM**	15	5	2	68.2%
**July 8 AM**	14	1	0	93.3%
**July 8 PM**	20	4	0	83.3%
**July 15 AM**	15	2	0	88.2%
**July 15 PM**	19	3	0	86.3%
**July 22 AM**	14	0	0	100%
**July 22 PM**	14	6	0	70%
**Overall response rate**	336	61	13	82%

**Table 2:** Demographic characteristics of study sample

**Participants by role**	**Unique respondents**	**Participants that responded on more than one survey administration**	**Mean surveys per respondent**	**Total # surveys**
**Attending MD**	10	3	1.9 (2.18)	19
			Min=1,Max=8	
**Resident MD**	18	6	1.3 (0.5)	24
			Min=1,Max=2	
**Nurse**	42	36	4.3 (2.1)	179
			Min=1,Max=9	
**HUC**	6	5	5.2 (3.4)	31
			Min=1,Max=10	
**PCT**	16	11	3.1 (2.0)	49
			Min=1,Max=10	
**Registration**	11	9	3.1 (1.6)	34
			Min=1,Max=5	
**Overall**	103	70	3.3 (2.3)	336
			Min=1,Max=10	
Mean Age	34.8 (11.2)	35.2 (11.4)	---	---
	Min=20,Max=60	Min=20,max=60		
Mean Years of Experience in this ED	5.3 (5.9)	4.9 (5.7)	---	---
	Min=0,Max=23	Min=0.08,max=23		
Mean Years of Experience in Healthcare	11.4 (9.9)	11.1 (9.8)	---	---
	Min=0,Max=35	Min=0,max=35		
Level of Education			---	---
High school or less	5 (5.0%)	4 (5.7%)	---	---
Some college, Undergraduate or Associate’s degree	64 (63.4%)	54 (77.4%)	---	---
Graduate School (i.e. Master’s, PhD, DrPH, or other)	5 (5.0%)	3 (4.3%)	---	---
Medical School (e.g. MD, DO)	27 (26.7%)	9 (12.9%)	---	---

**Figure 2:**
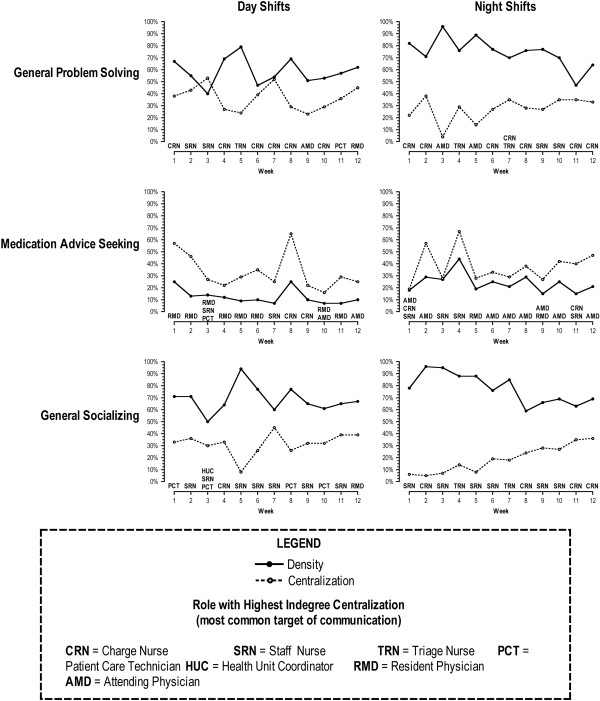
**Measures of density, centralization, and In-degree centralization by type of communication and over time.** The graph also highlights the role of the clinician with highest In-Degree centralization on the X-axis.

We observed wide variation in measures of network density across time points for all three measures of team communication (Figure 2). Values of network density were greatest for the day shift on week 5 for team communication related to general problem solving and social issues while it was highest on week 8 for the day shift for communications related to medication advice. (The value of network density for communications related to general social problem advice and social issues attained a second peak on week 8). We observed that across time points and shifts, network density was consistently lower for medication advice seeking communications than it was for communications for general problem solving advice and social issues. Figure 2 illustrates that, over time, network centralization values for medication advice seeking communication exceeded values of network density, whereas this was not the case for communication dealing with social issues or general problem solving. This implies that communication on medication related issues involved fewer teammates than did team communication on social issues or general problem solving. We also observed that values for network density and network centralization were different for day versus night shifts (Figure 2). Values of network density among night shift clinicians were greater than those for day shift clinicians across all three measures of communication and time points.

**Figure 3:**
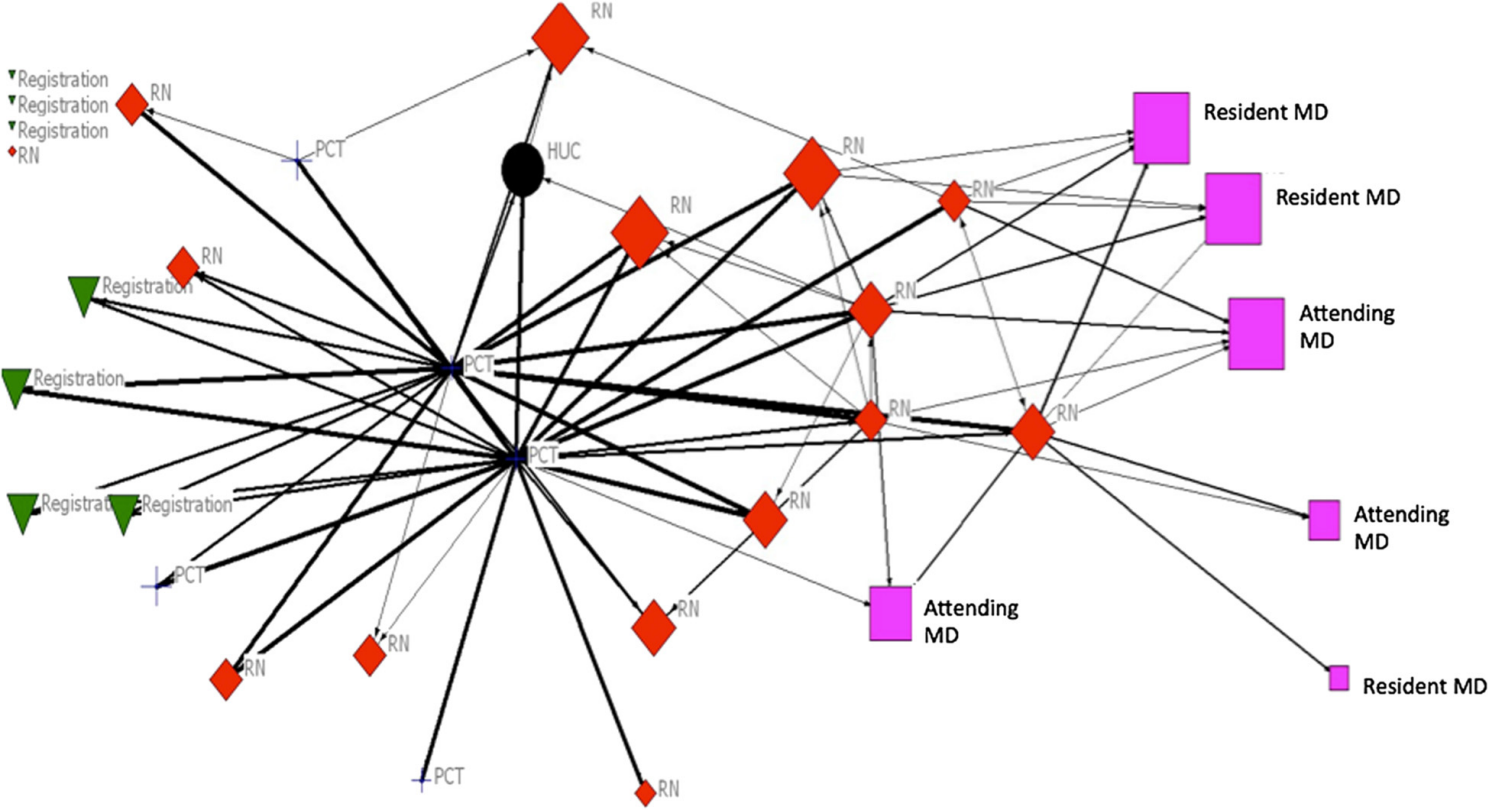
Sociogram of medication-advice seeking comminication during day shift and week eight of the study period.

We observed high values of in-degree centrality. These values imply that one or a select few individuals were the most frequently targeted – were the most common recipients of communication in the ED by teammates [38]. In this study, high in-degree communication values were most commonly linked to the charge nurse (CRN), staff nurse (SRN), and triage nurse (TRN) for general problem solving and general socializing communication (Figure 3). The most common target or recipient of team communication related to medication advice was the resident physician (RMD) and attending physician (AMD). These findings are consistent for both day and night shifts. We detected the highest in-degree centrality for the day shift RMD, SRN, and patient care technician (PCT) on week three for medication advice seeking communication.

Figure 3 is an example SNA sociogram that provides a graphic illustration of medication advice seeking communication between teammates on the day shift during week eight of the study. We chose this week for the sociogram because values for density and network centralization were greatest at this time in the study, offering the opportunity to visualize more communication ties between teammates. The thickness of the line between two teammates is based on frequency of communication. Thicker lines indicate greater communication frequency. We weighted the individual’s icon (node) based on his/her in-degree centrality. Larger node size indicates greater in-degree centrality. Figure 3 shows four individuals positioned in the upper left corner unattached to any other teammate. These are referred to as isolates. Two PCTs and several nurses at the center of the sociogram have multiple communication ties with many other teammates that worked during that shift period. These individuals are referred to as “stars,” who are often viewed as powerful, influential, or critical to the flow of information in an organization. In this study, in the context of communication, the sociogram shows that valuable information about medication was most frequently exchanged between two PCTs and several nursing staff. The sociogram does not provide data on the exact content of communication or whether or not the correct information was exchanged between teammates. The sociogram provides a high level view of communication ties between teammates and indication of whether or not communication is somehow constrained, focused, potentially lost, or disproportionate between teammates.

**Figure 4:**
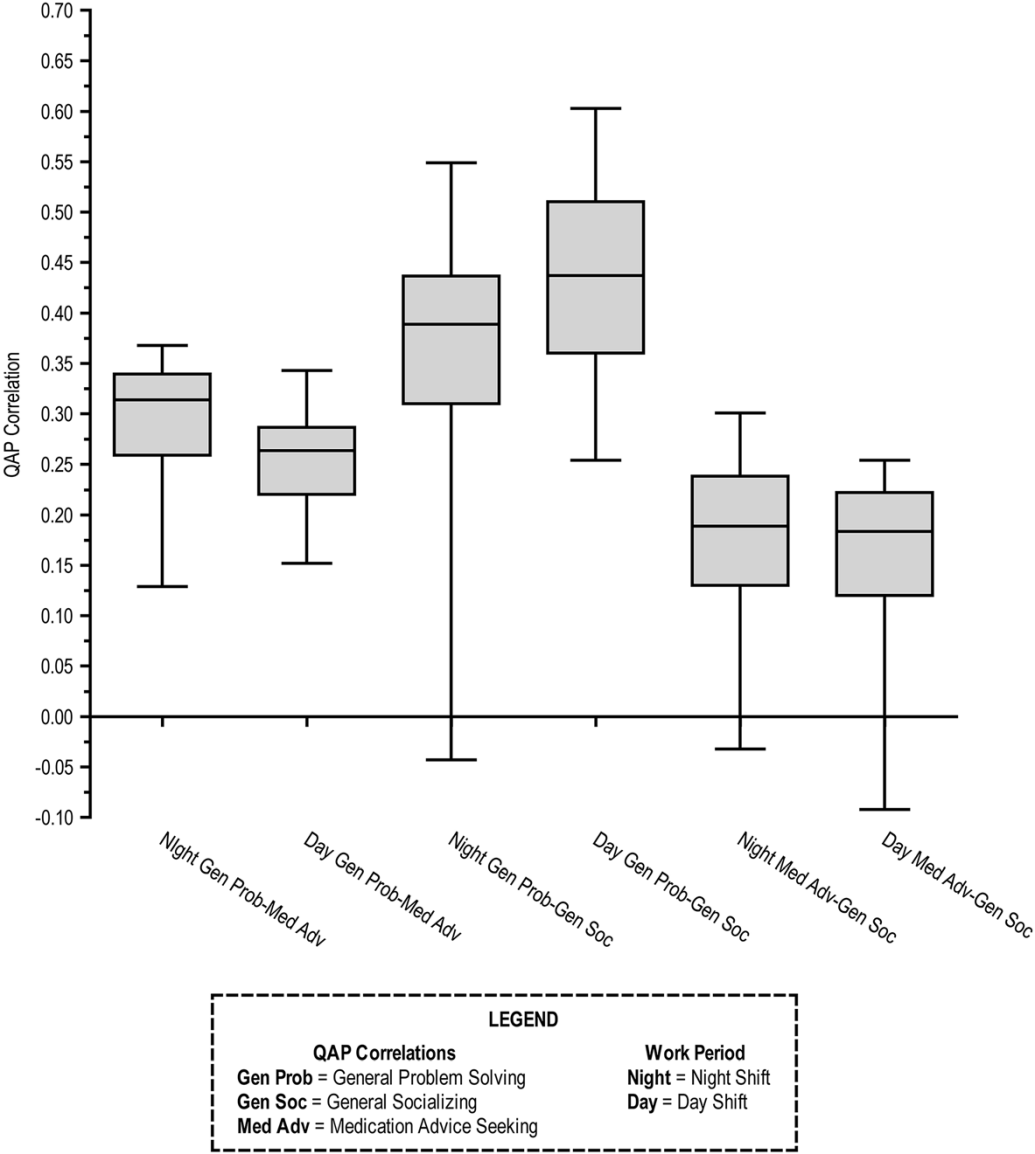
Boxplot of QAP correlations between communication networks, illustrating median, IQR, and minimum and maximum values.

Figure 4 displays the distribution of bivariate correlations between pairs of relations across the daytime and night shifts for all 12 weeks, for a total of 24 QAP correlations for each pair of network relations. The first two boxplots (from left to right) show the distribution of correlations between general problem solving and medication advice seeking; the first boxplot applies to people in the evening shift, and the second applies to people in the dayshift. What we observe here is a consistent but moderate correlation between these two relations over the course of the study in both the night and dayshifts. The majority of these correlations were between 0.2 and 0.37, and all but two of the 24 time periods were significant (per the QAP test). That is, there was a tendency for ED clinicians to approach other ED clinicians for advice on medications with whom they also approached for help on general problem solving.

The next two boxplots in Figure 4 show the distribution of correlations between general problem solving and general socializing communication networks. While these correlations are stronger, they are still only moderate in size. Most correlations were between 0.3 to 0.5, with the median correlation for the night shift and day shift being 0.38 and 0.43, respectively. All the correlations were significant except for one evening shift outlier (r= −0.04). That is, ED clinicians tended to approach the same people for social communication and for problem-solving communication.

The last two boxplots in Figure 4 display the distribution of correlations between general socializing and medication advice seeking. These correlations were the weakest. The majority of these correlations ranged from 0.12 to 0.26. Approximately half of the correlations (14 out of 24) were significantly different from 0 (per the QAP test). That is, while there was a slight tendency to approach those whom one socializes with for medical advice, this relationship was weak and often non-significant in these data.

## Discussion

The ED is staffed by a multi-disciplinary team of residents, interns, patient care technicians, health unit coordinators, and non-clinicians that work as a team to deliver care to the acutely ill. Our use of SNA provides a unique full-view of variation in communication in the ED and how all teammates are - or are not - communicating. We used SNA statistics to reveal variation in patterns of team communication and teammate interconnectedness over time and by shift. We demonstrated use of SNA statistic in-degree centrality as a measure of communication load by clinician role and uncovered variation in this measure over time and by shift, by presenting a graph that exposed dramatic intensifications and reductions between measurements. We also identified redundancy in relations, with communication of general problem solving interactions being related to both socializing communication and medication advice seeking communication (Figure 4). However, there are enough differences among these relations to warrant considering each separately as one investigates patterns of communication and coordination in the ED setting.

These findings contribute to a previously acknowledged complexity of communications research and support our belief that much about communications in the ED is unknown. We believe our findings challenge the notion that a one-time assessment of team communication accurately represents team communication patterns of future shifts. One-time assessments may over or under estimate the true nature and complexity of team communication in team relationships. Such findings can be detrimental to the success of interventions developed, at least in part, on one-time assessments.

While limited to brief periods of assessment, we identified some similarities between our findings and the results of previous research. A study by Fairbanks and colleagues used link analysis and brief periods of direct observation to examine variation in communication load and direction of communication between physicians and nurses [24]. The observers in this study documented frequent communication between physicians and nurses, but weak communication (infrequent) by other types of clinicians that interacted with the clinicians being observed [24]. A separate study by Coiera and associates determined that communication in the ED most often originates with a nurse and ends with a physician rather than in the opposite direction [11]. Our findings for the medication advice seeking communication measure agree with these observations, though we reached different conclusions with respect to communication that addressed general problem solving or social communication. Social scientists stress that awareness of frequent targets for different types of communication can be useful for identifying individuals that affect the dissemination of information and adoption or rejection of policies in the workplace [29]. These individuals wield considerable power in the workplace and are often unnoticed or not easily identified on an organizational chart or with techniques that fail to consider the “network” of workers as a whole.

Our results expand findings of prior research by Creswick and colleagues who used SNA to quantify the structural characteristics of communication between ED teammates [23]. In their study, the estimated network density for socializing communication (18%) was lower than our estimate across all time points and across day and night shifts (Day shift range 50–94, Night shift range 59–96, see Figure 2) [23]. Our weekly measures of network density for problem-solving communication indicated a greater level of cohesion of communication between teammates than estimates from the study by Creswick and colleagues, estimating density at 53% (Day shift range 40–69, Night shift range 47–96). Finally, our measures of density for medication advice seeking communication were consistently lower than that identified previously at 37% (Day shift range 7–25, Night shift range 15–44). We feel these differences are attributed to the differences in study design between our respective studies. Creswick and colleagues used a one-time assessment and we used multiple assessments across day and night shift. These differences highlight the need to investigate communication patterns over time and across shifts to capture a more accurate signal of in team communication in the ED setting.

Poor team communication is the most common root cause of sentinel events (errors) in healthcare [2-4]. Improving communication is a key objective of safety-focused programs for healthcare (i.e., Crew Resource Management (CRM) and TeamSTEPPS) [44]. These programs emphasize frequent, clear, and closed loop communication between all teammates [45,46]. Our findings highlight a concentration of communication activity between groups or cliques of teammates. Not all teammates are communicating and the frequency or amount of communication between those that do communicate is unequal. Our findings pose several questions; 1) Do all teammates in the ED need to communicate during shiftwork? 2) Would lack of communication between all teammates post intervention be considered a failure? Answers to these questions are absent given our limited understanding of communication in the ED and its impact on outcomes.

We determined that research on ED team communication has yet to characterize communication between all teammates and describe the amount and pattern of communication that contributes to poor or positive outcomes. Research of communication patterns over time and between all teammates is needed and critical because of the reasons discussed above and because healthcare teams are increasingly multi-disciplinary. The assembly of diverse practitioner pools is accompanied by the expectation that each individual will do his or her best to work as a team, communicate openly, frequently, in a closed-loop manner with all teammates to prevent medical error and adverse events [47]. Are our expectations of our clinicians likely to be met given the limitations of the current research and our uncertainty of communication behaviors in the ED setting? The next effort should begin with the aim to disentangle the complexity of communication in the ED.

A key limitation of prior research is brief periods of observation of a select few clinicians (e.g., physicians or nurses) [11]. We found that SNA can quantify patterns of communication between all clinicians and provide a comprehensive and more accurate depiction of communication interdependencies. We believe SNA is appropriate for investigating communication in the ED as a whole (network wide) because the connections/interdependencies between all clinicians are captured [29]. Moreover, we believe that SNA is an efficient approach to collect this information; we achieved a high participation rate, which we believe is a reflection on the ease associated with administering short SNA surveys.

The operational and demographic characteristics of our selected study environment may not generalize to other settings. Sex differences may impact communication between clinician-patient and clinician-clinician communication [48]. We did not capture sex and cannot assess its impact. We used cross-sectional surveys that can suffer from recall bias, though we minimized this by administering our surveys at the end of each targeted shift. Variation in how respondents interpreted the meaning of each communication measure may impact our findings by increasing or decreasing the values of network density, centralization, or in-degree centrality. We addressed this threat by 1) formatting our SNA surveys and items to be consistent with standard SNA survey techniques, and 2) by training our research assistant to offer standard clarification on the meaning of survey items if questioned [27,28,37,49,50].

We demonstrate the potential value of SNA as a tool for communications research in a high-risk clinical setting. Focus groups or participant interviews may provide additional insights regarding the implications of our SNA findings. The eventual value or utility of SNA requires tying measures of density and centralization to clinical process measures or outcomes such as the number of patients leaving without being seen or medical errors.

## Conclusions

We show that SNA techniques aid in developing a systems-level view of clinician-to-clinician communication in the ED setting. In our study sample, SNA measurement techniques reveal that the frequency of communication as a measure of interdependence between ED clinicians varies by day/night shift and over time.

## Competing interests

The authors have no competing interests. This work was supported by grants from the American Society for Healthcare Risk Management (http://www.ashrm.org) and Pittsburgh Emergency Medicine Foundation (http://www.pemf.net). Dr. Patterson is supported by a career-training award (Grant Number *8KL2TR000146-07*) from the National Center for Research Resources (NCRR), a component of the National Institutes of Health (NIH), and NIH Roadmap for Medical Research. The contents of this article are solely the responsibility of the authors and do not necessarily represent the official view of NCRR or NIH. Information on NCRR is available at http://www.nih.gov/about/almanac/organization/NCRR.htm.

## Authors’ contribution

The study was conceptualized by PDP, DK, RMA, DMY, and JRL. Authors PDP, AJP, and MDW were responsible for study deployment and data collection. Authors PDP, AJP, DK, and MDW performed the analysis of study data. Findings were reported in the manuscript and the manuscript edited and approved by all authors PDP, AJP, MDW, DK, RMA, DMY, and JRL.

## Prior conference presentations

Portions of this work were presented at the 2010 American Society for Healthcare Risk Management (ASHRM) annual meeting in Tampa, Florida on October 16, 2010.

## Pre-publication history

The pre-publication history for this paper can be accessed here:

http://www.biomedcentral.com/1472-6963/13/109/prepub
